# Rib Cortical Bone Fracture Risk as a Function of Age and Rib Strain: Updated Injury Prediction Using Finite Element Human Body Models

**DOI:** 10.3389/fbioe.2021.677768

**Published:** 2021-05-24

**Authors:** Karl-Johan Larsson, Amanda Blennow, Johan Iraeus, Bengt Pipkorn, Nils Lubbe

**Affiliations:** ^1^Autoliv Research, Vårgårda, Sweden; ^2^Division of Vehicle Safety, Department of Mechanics and Maritime Sciences, Chalmers University of Technology, Gothenburg, Sweden

**Keywords:** rib fracture, injury risk, injury prediction, human body model, occupant safety, survival analysis, SAFER HBM

## Abstract

To evaluate vehicle occupant injury risk, finite element human body models (HBMs) can be used in vehicle crash simulations. HBMs can predict tissue loading levels, and the risk for fracture can be estimated based on a tissue-based risk curve. A probabilistic framework utilizing an age-adjusted rib strain-based risk function was proposed in 2012. However, the risk function was based on tests from only twelve human subjects. Further, the age adjustment was based on previous literature postulating a 5.1% decrease in failure strain for femur bone material per decade of aging. The primary aim of this study was to develop a new strain-based rib fracture risk function using material test data spanning a wide range of ages. A second aim was to update the probabilistic framework with the new risk function and compare the probabilistic risk predictions from HBM simulations to both previous HBM probabilistic risk predictions and to approximate real-world rib fracture outcomes. Tensile test data of human rib cortical bone from 58 individuals spanning 17–99 years of ages was used. Survival analysis with accelerated failure time was used to model the failure strain and age-dependent decrease for the tissue-based risk function. Stochastic HBM simulations with varied impact conditions and restraint system settings were performed and probabilistic rib fracture risks were calculated. In the resulting fracture risk function, sex was not a significant covariate—but a stronger age-dependent decrease than previously assumed for human rib cortical bone was evident, corresponding to a 12% decrease in failure strain per decade of aging. The main effect of this difference is a lowered risk prediction for younger individuals than that predicted in previous risk functions. For the stochastic analysis, the previous risk curve overestimated the approximate real-world rib fracture risk for 30-year-old occupants; the new risk function reduces the overestimation. Moreover, the new function can be used as a direct replacement of the previous one within the 2012 probabilistic framework.

## Introduction

Despite improvements in vehicle occupant safety (Kullgren et al., 2019), rib fractures remain a prevalent outcome in motor vehicle collisions (MVCs) (Forman et al., 2019; Pipkorn et al., 2020). Among patients admitted to emergency care for blunt chest trauma, MVCs are the major cause of injury; moreover, having three (or more) fractured ribs is a risk factor for mortality (Sirmali et al., 2003; Veysi et al., 2009; Battle et al., 2012). Epidemiological studies reveal that risk of thoracic injury, including rib fractures, in MVCs increases with impact speed and age—and is greater for females than for males (Bose et al., 2011; Carter et al., 2014; Weaver et al., 2015; Brumbelow, 2019; Forman et al., 2019). Increased impact speed increases the energy (and concomitant mechanical load) transferred to the occupant’s thorax from vehicle safety systems, e.g., seatbelts. The increased rib fracture risk with age can be partly explained by findings from studies of human bone’s mechanical properties, which show that tolerance to mechanical load until fracture decreases with age (Lindahl and Lindgren, 1967; Burstein et al., 1976; Carter and Spengler, 1978; McCalden et al., 1993; Kemper et al., 2005). Among these studies, Kemper et al. (2005) reported a difference in bone’s ultimate strain due to sex, with the females showing reduced deformation before failure, but here the three female bone material donors were on average older than the three male donors, suggesting that the noted reduction may have been an effect of age rather than sex (Kemper et al., 2005). McCalden et al. (1993) reported a small increase in ultimate stress for female femoral specimens, while Lindahl and Lindgren (1967) and Burstein et al. (1976) did not find any significant differences in femoral cortical bone ultimate stress between the sexes.

In order to design safer vehicles, it is necessary to have tools and methods that can predict the influence of design changes on injury outcome. Finite element human body models (HBMs) are used in vehicle crash simulations to estimate occupant injury risk, including rib fracture risk, and to evaluate and develop countermeasures. The injury risk can be estimated using local tissue measurements, such as stress and strain in the modeled anatomical structures. Rib cortical strain has been shown to correlate to fracture in postmortem human subject (PMHS) tests (Trosseille et al., 2008). One commonly used HBM which has been validated for predicting strain in the rib cortical bone for various impact loads is the SAFER HBM (Iraeus and Pipkorn, 2019).

An injury risk function is necessary to establish a mathematical link between rib cortical strain and rib fracture risk. A variety of statistical methods have been employed in the past to create injury risk functions, as described in Petitjean and Trosseille (2011). Commonly used are logistic regression and survival analysis. While the resulting injury risk curves can differ substantially depending whether exact or censored data are used (Praxl, 2011), in most situations the two methods produce similar results (McMurry and Poplin, 2015). Petitjean and Trosseille (2011) recommend survival analysis, based on statistical simulations of theoretical samples. In addition, the International Organization for Standardization (ISO) proposed a 12-step approach to constructing injury risk curves from PMHS testing using survival analysis (International Organization for Standardization, 2014). This approach was applied to thoracic risk curves for WorldSID (Petitjean et al., 2012) and THOR (Davidsson et al., 2014).

A probabilistic framework detailing how to translate injury risk for an individual rib as calculated by injury risk functions (developed using survival analysis or otherwise) to a risk of sustaining a certain number of rib fractures in HBM simulations was presented in 2012 (Forman et al., 2012). The framework included also a specific rib cortical bone strain-based injury risk function which was based on dynamic test data from twelve human subjects (Kemper et al., 2005, 2007). The data were biased towards older subjects (only one subject was below the age of 42). Age adjustment was performed by assuming a reduction in rib cortical bone failure strain of 5.1% per decade of aging, based on test data of femur cortical bone reported by Carter and Spengler (1978). The 5.1% reduction had been originally reported by Burstein et al. (1976) from testing of material from *N* = 33 donors (21–86 years old); they also reported a 6.9% reduction in failure strain per decade of aging for tibial cortical bone samples (*N* = 28, 21–86 years old). McCalden et al. (1993) reported that the reduction of failure strain in femoral cortical bone was 9% per decade (*N* = 47, 20–102 years). Thus, Forman et al. (2012) created the risk function in the framework using relatively few, predominantly older subjects and applied a relatively small age-dependent decrease. The original risk function from Forman et al.’s (2012) study (referred to hereafter as “Forman 2012”) was not based on survival analysis but presented as an empirical cumulative distribution function. The drawback with this type of function is that very small strain increments can give large risk increments, which is an undesired feature in design optimization. To overcome this limitation, the framework was updated with a smooth risk curve (Iraeus and Lindquist, 2020). The same Kemper et al. (2005); Kemper et al. (2007) strain data and 5.1% reduction used for the Forman 2012 risk curve was used, but a Weibull distribution was fitted. The resulting risk function will be referred to as “Forman smoothed”.

Rib fracture risk predictions from the probabilistic framework, updated with the Forman smoothed risk curve, were validated against rib fractures observed in field data by Pipkorn et al. (2019). The rib strains used as input for the probabilistic risk calculation were obtained from the SAFER HBM. Detailed accident reconstructions and population-based stochastic vehicle impact simulations were performed. The predicted risk increased with impact speed as expected, but for younger occupants the framework overestimated the rib fracture risk at any given impact speed even more than for elderly occupants. This risk overestimation is likely a consequence of the low age-dependent decrease in the “Forman smoothed” rib strain risk function.

Recently, material coupon tensile testing was performed on human rib cortical bone samples from 61 PMHSs (32 males and 29 females) ranging in age from 17 to 99 years (Katzenberger et al., 2020). These data suffice to develop an age-dependent rib strain-based fracture risk function without relying on age scaling from other sources.

The aim of this study was to develop a strain-based rib fracture risk function using material test data spanning a wide range of ages. The influence of age and sex on the fracture risk was investigated and modeled. A second aim was to update the probabilistic framework with the new risk function and compare probabilistic risk predictions from a set of existing HBM simulation rib strain results. The updated predictions were compared to previous predictions obtained using the Forman smoothed risk function.

## Materials and Methods

### Materials

The data used in this study have previously been presented by Katzenberger et al. (2020). The authors reported mechanical properties of human rib cortical bone, measured by tensile testing of samples from PMHSs. From each PMHS, two coupons of rib cortical bone were extracted from rib levels 3 to 7. The coupons were subjected to uniaxial tensile tests to failure at medium or low strain rates (0.5 and 0.005 strain/s, respectively). The higher rate was selected to represent the strain rate measured on PMHS ribs in experiments simulating a 48 km/h frontal impact (Duma et al., 2005; Katzenberger et al., 2020). Results were obtained and reported from 58 medium-rate tests and 58 low-rate tests (55 medium and low rate test results from the same PMHS). The age and sex of the PMHSs and the reported strain at which the samples failed (failure strain, reported as engineering strains, i.e., sample elongation at failure divided by initial length) comprise the data used in this study.

### Rib Fracture Risk Function

The method for developing the new rib cortical bone fracture risk function follows the 12-step procedure for developing injury risk curves, according to International Organization for Standardization (2014) and Petitjean et al. (2012): (1) collect data, (2) assign censor status, (3) check for multiple injury mechanisms, (4) separate samples by injury mechanism, (5) estimate distribution parameters, (6) identify overly influential observations, (7) check the distribution assumption, (8) choose the distribution, (9) check the validity of predictions against existing results, (10) calculate 95% confidence intervals, (11) assess the quality index, and (12) recommend one curve per body region.

In Step 1, age, sex, and failure strain of each donor PMHS in the 0.5 strain/s experiments were selected (one sample per PMHS). As the resulting fracture risk function is intended for use with HBM strains obtained in vehicle impact simulations, it was assumed that the higher strain rate will be applicable for injurious impacts. Censoring status was assigned as exact for all failure strain values (Step 2). There was no indication of more than one failure mechanism in this controlled testing (Steps 3 and 4). Thus, the collected data for creation of the injury risk curve consist of failure strain, age, and sex from 58 PMHS (31 males and 27 females). Ages ranged from 17 to 99 years (mean 56.2 years; SD 26.1). The failure strain values were then recomputed from engineering to true strain (also known as logarithmic strain) values, to correspond to the format used with explicit finite element codes.

The available data were analyzed to select relevant covariates. An ANOVA test (R software v.3.6.3; stats package v.4.0.2) (R Core Team, 2020), was used to determine whether age and sex significantly influence the failure strain (in which case they should be modeled as covariates). Survival analysis was used to calculate the probability of survival [R; flexsurv package v.1.1.1 (Jackson, 2016)], in order to model the risk of rib material fracture as a function of failure strain and covariates. The probability of fracture was then computed as 1-(probability of survival). Upon inspection, the failure strain appeared to decrease log-linearly with age, hence an accelerated failure time (AFT) model was used. Log-normal, log-logistic, and Weibull distributions were considered for the parametric AFT model formulation, and the parameters were estimated with the maximum-likelihood method (Step 5).

In Step 6, the method of DFBETAs, with a threshold of 2 divided by the square root of sample size, was used to identify any overly influential data points (Belsley et al., 1980). The distribution assumptions were checked using Q–Q plots (Step 7). Tukey-Anscombe plots of model residual versus fitted values were checked.

For Steps 8–12, 95% confidence intervals for the survival curve were determined assuming an asymptotic normal distribution. The Akaike information criteria (AIC) and Quality indices (QIs) were computed for each of the log-normal, log-logistic, and Weibull distributions. QIs were computed based on the relative size of the confidence interval (Petitjean et al., 2012) at 5, 25, and 50% risk, for the ages 25, 50, and 75 years. The resulting risk functions were visually compared to the Forman 2012 and Forman smoothed risk functions. Finally, a single risk function was chosen for strain-based rib fracture risk based on QIs and the AIC values.

### Population-Based Simulations to Quantify Effect on HBM Risk Predictions

To evaluate the effect of the newly developed strain-based risk function for a population of vehicle crashes and occupants at different ages, the stochastic simulations in Pipkorn et al. (2019) were reanalyzed by re-computing (using the newly developed rib fracture risk function) the probabilistic rib fracture risk using the rib strains from each of the stochastic simulations in Pipkorn et al. (2019). No new simulations were performed in this study. The method, including the National Automotive Sampling System Crashworthiness Data System (NASS/CDS) reference risk curves, is described in detail in Iraeus and Lindquist (2016) but is briefly described here. Two datasets from the NASS/CDS database were defined, one including frontal crashes (first analyzed in Iraeus and Lindquist, 2016) and one including side impacts (first analyzed in Pipkorn et al., 2019). Both datasets included both injured and uninjured occupants. Frontal crashes were selected based on NASS/CDS variable GAD1 = “F” and near-side impact were selected based on GAD1 = “L” (for drivers) or “R” (for front seat passengers). Other inclusion criteria were; NASS/CDS case years 2000–2012; vehicle model year 2000 or later (MY 2000+); the vehicle should had a deployed airbag (steering wheel airbag for drivers or passenger airbag for front seat passengers in frontal impacts, and side airbag in side impacts); and the occupant should be an adult belted front-seat occupant (AGE 17+). Rollovers were excluded (ROLLOVER = 0). The set of frontal impact crashes contained 5,083 cases (1,474,869 cases weighted—i.e., representing national prevalence according to NASS/CDS national inflation factors), with 185 occupants (17,810 occupants weighted) sustaining two or more fractured ribs (NFR2+). The set of side impact crashes contained 569 cases (166,209 cases weighted), with 60 occupants (3,495 occupants weighted) sustaining a NFR2+ injury. Injury risk curves were created using weighted logistic regression (R software, version 3.6.3; survey package v.4.0). Occupant age and NASS/CDS-estimated change in velocity (Delta-v, as calculated by WinSmash) were considered as covariates. In the original analysis (Iraeus and Lindquist (2016)) vehicle instrument panel intrusion was also found to be a significant covariate. However, when compared to the simulations the intrusion was set to zero (both in the NASS/CDS regression model and in the simulations). Sex was also tested as covariate but was not significant (*p* = 0.92).

**FIGURE 1:**
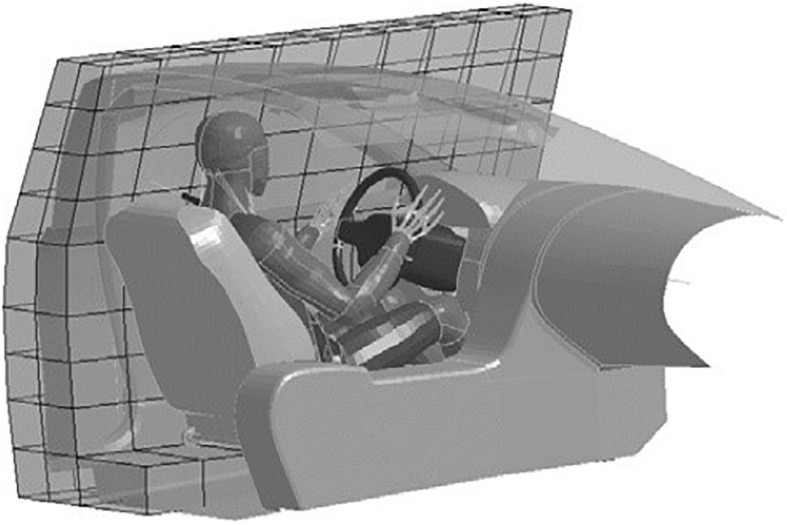
SAFER HBM version 9 and the parametrized vehicle interior model used to estimate rib fracture risk in a population of NASS/CDS crashes. The coarse grid is used to apply the lateral velocity profile to the side structure.

Next, two stochastic simulation studies, one frontal and one lateral, were defined as described in Pipkorn et al. (2019). For both studies, the SAFER HBM version 9 (Iraeus and Pipkorn, 2019; Pipkorn et al., 2019) was positioned in a parameterized finite element model of a vehicle interior (Iraeus and Lindquist, 2016). For frontal impacts, the vehicle model included a driver airbag, a load-limited seat belt, and dashboard and floor pan intrusion modeling. For the lateral impacts, side impact countermeasures (Pipkorn et al., 2019) and side structure intrusion modeling (Figure 1) were added. Using Latin Hypercube sampling, the vehicle and crash pulse parameters were varied according to distributions from the NASS/CDS datasets. The study consisted of 1,000 frontal impact simulation models and 100 lateral impact simulation models. More details about the method can be found in Iraeus and Lindquist (2016).

For each simulation, the NFR2+ risk was analyzed, using the probabilistic rib fracture framework with two different rib fracture risk functions: Forman smoothed and this study’s newly developed risk function. In each case, the input to the probabilistic framework was the same peak first principal strains from each of the 24 rib cortical bone meshes in the HBM, extracted from each impact simulation. For both sets of results, quasi-binominal regression was used to create population risk curves, which were then compared to the NASS/CDS population risk curves.

## Results

### Rib Fracture Risk Function

The ANOVA showed that age had a significant effect on (true) failure strain (*p* < 0.0001). Neither sex (*p* = 0.335) nor the interaction of sex and age (*p* = 0.187) were significant as predictors for failure strain at the α = 0.05 significance level; they were thus excluded as covariates (for the complete ANOVA analysis output, see Appendix Table B1).

The DFBETAS statistics highlighted six failure strain and age observations from the sample as potentially overly influential. For each of these observations, the experimental stress-strain curve was visually compared to the stress-strain curves of other observations of similar age. No differences (such as very low or high failure strain, measurement signal noise, or differences in stress magnitude) could be identified. All the highlighted observations were therefore kept in the sample.

**FIGURE B1:**
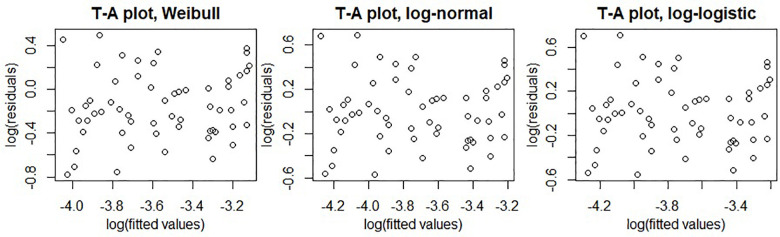
Tukey-Anscombe plots of residuals versus fitted values for fitted Weibull **(left)**, log-logistic **(middle)**, and log-normal **(right)** survival models.

**FIGURE B2:**
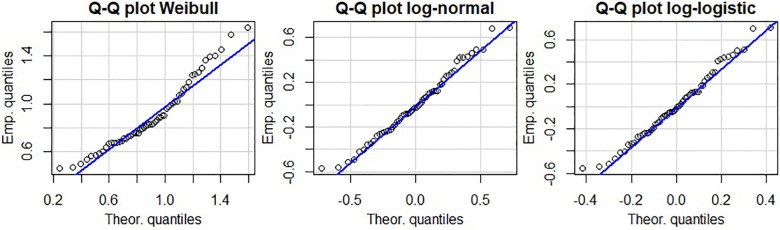
Q–Q plots of residuals versus fitted Weibull **(left)**, log-logistic **(middle)**, and log-normal **(right)** distributions.

Injury risk was computed following a parametric AFT survival model with the alternatives of log-normal, log-logistic, and Weibull distributions. The distribution’s parameters are presented in Table 1. Parametric fracture risk expressions for each distribution are given in Appendix A. Tukey-Anscombe plots showed no evident trends for the residuals (Appendix Figure B1). Q–Q plots of survival model residuals versus each distribution did not reveal any systematic violations of distribution assumptions (Appendix Figure B2).

**TABLE 1:** Distribution parameters for Weibull, log-normal, and log-logistic distributions.

**Distribution**	**α**	**β_0_**	**β_1_**
Weibull	3.3562	−2.9236	−0.0114
Log-normal	0.3026	−2.9866	−0.0130
Log-logistic	5.6986	−2.9802	−0.0133

**FIGURE 2:**
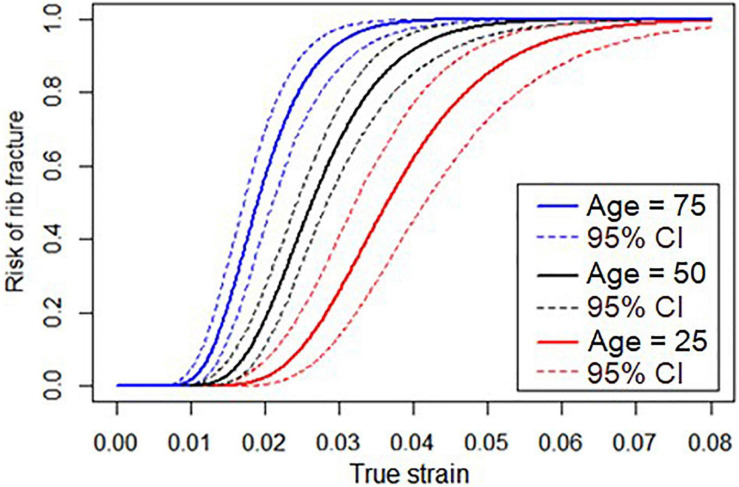
Risk function relating true strain and risk of rib fracture for subjects who are 25, 50, and 75 years old.

All distributions obtained good QIs, given the confidence interval sizes (Appendix Table B2), so the QIs could not be used to select the best model fit. The selection was therefore based on the lowest AIC value. The lowest AIC value, AIC_*min*_ = −399.30, was obtained for the log-normal distribution. Weibull and log-logistic distributions obtained AIC values of −389.50 (AIC_*mi*__*n*_ + 9.80) and −397.09 (AIC_*min*_ + 2.21), respectively. Therefore, the recommended risk function for rib fracture based on strain and age is modeled with the log-normal distribution. The parametric expression of the recommended rib fracture risk function, based on the log-normal distribution, is given in Eq. 1.

$$Fracturerisk\left(strain,AGE\right)=\frac{1}{2}$$

(1) $$+\frac{1}{2}\mathrm{erf}\left[\frac{\mathrm{LN}\left(\mathrm{strain}\right)-\left(\beta_{0}+\beta_{1}\cdot \mathrm{AGE}\right)}{\sqrt{2}\cdot \alpha }\right]$$

where α, β_0_, and β_1_ can be found in Table 1 for log-normal distribution parameters. LN() is the natural logarithm and erf() is the Gauss error function. The resulting risk function, relating strain and age to the risk of fracture, is plotted in Figure 2 for subjects who are 25, 50, and 75 years old.

**FIGURE 3:**
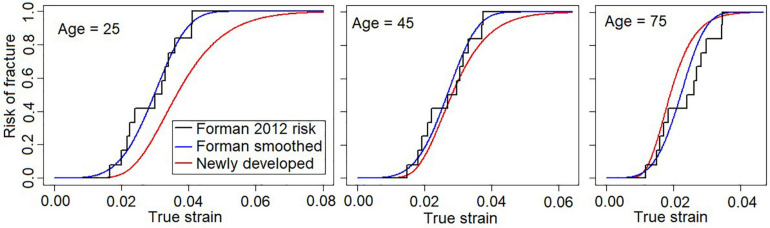
Newly developed risk function (red) with Forman 2012 (black), and Forman smoothed (blue) functions for three different ages. **Left**, 25 years; **middle**, 45 years; **right**, 75 years.

The recommended risk function (further referred to as the “newly developed”) is compared to the previously existing risk functions, Forman 2012 and Forman smoothed, in Figure 3 for three different ages. For the oldest individuals (75 years), the new risk function predicts slightly higher fracture risks than the previous risk functions. As an example, for the new risk function, a rib strain value of 0.02 is associated with 56% fracture risk for a 75-year-old, while for the Forman 2012 and Forman smoothed risk functions, the risk is approximately 40%. For 45-year-olds, the risk predictions are similar, while for the 25-year-olds, the newly developed risk function predicts lower risk.

### Population-Based Simulations to Quantify Improvement of Risk Curves

**FIGURE 4:**
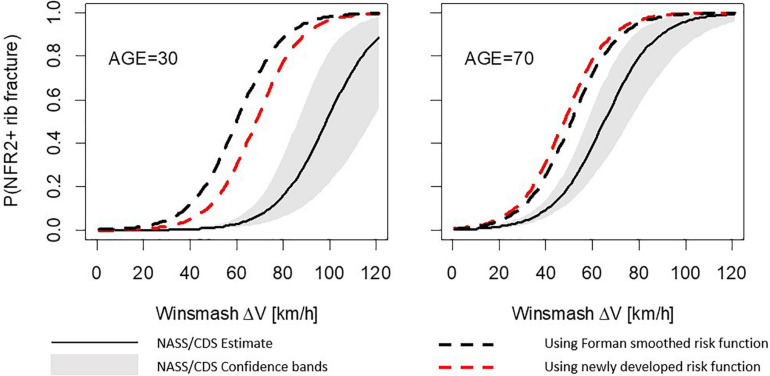
Number of fractured ribs, NFR2+; predictions for the frontal stochastic simulations. Comparison between risk curves from probabilistic framework using the Forman smoothed and the newly developed risk functions for a 30-year-old occupant **(left)** and a 70-year-old occupant **(right)**.

For the frontal load case, using either the Forman smoothed risk function or the newly developed risk function within the probabilistic framework, the simulation model demonstrates a higher NFR2+ risk than the NASS/CDS risk curves, regardless of occupant age; see Figure 4. However, the distance between the solid line (NASS/CDS estimated risk curve) and the dashed lines (simulation-based estimated risk curves) is more consistent over ages for the newly developed risk function. As an example, we examine the 50% risk: the probabilistic framework with the Forman smoothed risk function predicts 50% risk for a 30-year-old occupant at a Delta-v of 60 km/h; with the newly developed risk function, a 50% risk is predicted at a Delta-v of 69 km/h. The NASS/CDS estimate is 98 km/h, representing underestimations of 38 km/h and 29 km/h for the Forman smoothed and the new function, respectively. For a 70-year-old occupant, the corresponding underestimations of the Delta-v for the 50% NASS/CDS risk are 14 km/h (Forman smoothed) and 17 km/h (newly developed risk function). That, is, when comparing the risk for 30- and 70-year-olds, the differences between the NASS/CDS risk and the risk predicted by the simulation model are more consistent for the newly developed risk function.

**FIGURE 5:**
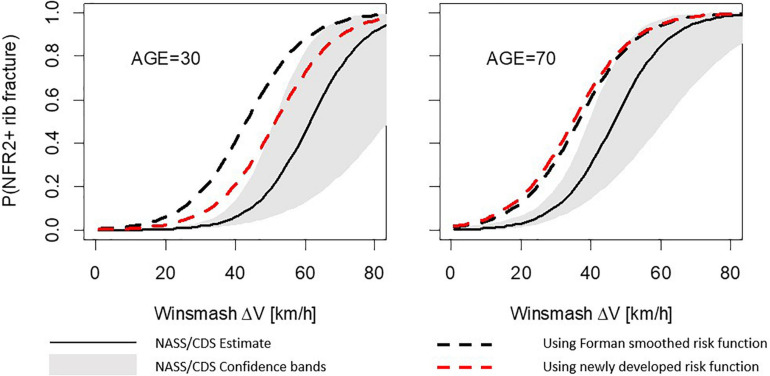
Number of fractured ribs, NFR2+; predictions for the lateral stochastic simulations. Comparison between risk curves from probabilistic framework using Forman smoothed and the newly developed risk functions for a 30-year-old occupant **(left)** and 70-year-old occupant **(right)**.

The results for the lateral load case show similar trends; see Figure 5. Using the Forman smoothed risk function, the 50% risk for a 30-year-old occupant is predicted at a Delta-v of 43 km/h, an underestimation of 18 km/h. Using the newly developed risk function it is predicted at a Delta-v of 52 km/h, an underestimation of 9 km/h. For a 70-year-old occupant, the corresponding underestimations of the Delta-v for the 50% NASS/CDS risk are 10 km/h (Forman smoothed) and 11 km/h (newly developed risk function). As for the frontal load case, the simulation model predictions using the new function for the lateral load case are closer to the NASS/CDS risk estimates, and partly within the confidence bands.

## Discussion

A new rib fracture risk function was developed using a parametric AFT survival model. AIC was used to select the log-normal distribution. It has been debated whether AIC is suitable for choosing the distribution, or if one should default to a Weibull distribution, or if the Area under the Receiver Operator Curve, indicating how good injury and non-injury data are classified, is a better metric (Yoganandan et al., 2016, 2017; McMurry and Poplin, 2017). For the developed risk curves the Weibull distribution performed worst in terms of AIC, but with an AIC delta of less than ten compared to the other distributions. As evidenced by the QIs being equally good for all distributions, there is no strong evidence against the Weibull distribution; however, there was no reason not to choose the log-normal distribution that had the lowest AIC value. Parameters for the Weibull distribution are reported in Table 1, should one prefer it. The dataset consisted of test-to-failure data only, hence, the Area under the Receiver Operator Curve cannot be calculated. Further details on choosing predictors of interest and identifying overly influential observations have been suggested (Yoganandan et al., 2016) and debated (McMurry and Poplin, 2017; Yoganandan et al., 2017). However, in the current data, no outliers were identified and the selection of predictors of interest was straightforward and based on previous literature, likely not requiring even more detailed analysis. Alternatives such as Bayesian survival analysis may offer improvements for small sample sizes (Cutcliffe et al., 2012), but with 58 tests the sample used is likely large enough for accurate estimations without it. Overall, the 12-step ISO approach appears to be a viable approach and well suited to the data in this study.

**FIGURE 6:**
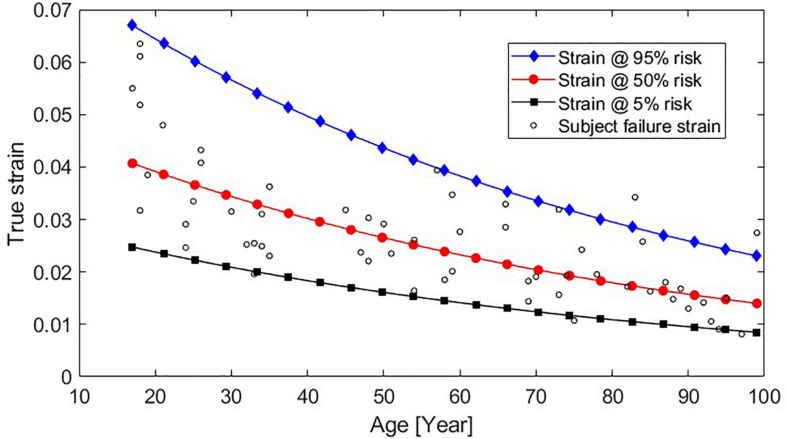
Strains that result in 95, 50, and 5% predicted fracture risk across subjects aged 17–99 years, using the newly developed function plotted with the failure strain and age data from each subject.

The age effect (the decrease in failure strain as a function of age) is greater for the newly developed risk function compared to the previous risk functions used with the probabilistic framework (Forman 2012 and Forman smoothed), see Figure 3. In the current study, the AFT model was used for the survival analysis, resulting in a proportional relationship between age and failure strain. The acceleration factor is exp(β_1_⋅*AGE*). Using β_1_ for the recommended log-normal distribution from Table 1, after 10 years of aging a subject will only require 87.8% of the strain to predict the same risk of fracture as before. In other words, according to our modeling, the failure strain in human rib cortical bone is reduced by 12.2% per decade of aging. This reduction appears greater than both the 5.1% reduction (Carter and Spengler, 1978) used in the Forman 2012 risk function and the 9% reported by McCalden et al. (1993). To investigate if the age-dependent decrease found in the current study is reasonable, we can compare the risk predictions from the newly developed function to the failure strains in the dataset used. In Figure 6, the strains required for 5, 50, and 95% risk predictions from the newly developed risk function across the 17–99 year age span are plotted with the age and failure strain of each subject. A visual comparison demonstrates that the strains representing a 50% risk level appear centered between the subject failure strains across the age span. In other words, for a given strain and age, a risk prediction of 50%, corresponds well to the expectation that half of the test samples of that age failed at that level of strain. Similarly, for the 5% risk level, we can expect that most, but not all, samples will survive that level of strain. Thus, the 12.2% reduction factor appears to be a reasonable estimation of the age-dependent decrease in the subject failure strains.

Sex was not found to have a significant effect on the failure strain; this result is in agreement with the findings in Katzenberger et al. (2020), where it was shown that sex did not have any statistically significant effect on any of the rib cortical bone material parameters, yield stress and strain, elastic modulus or failure stress, at either of the strain rates (0.5 and 0.005 strain/s). Sex was not included as a covariate (as it was not significant) in the NASS/CDS regression model, and the stochastic simulations were only carried out using a model of the average male. This result is in conflict with some epidemiological studies, Bose et al. (2011); Carter et al. (2014), and Forman et al. (2019) who found an increased rib fracture risk for females compared to males. However, it should be noted that the two covariates included in the current study, Delta-v and age, are the two most important parameters as they have the largest effect size. In Forman et al. (2019) sex has the same effect on rib fracture risk as changing Delta-v by 5.8 km/h or occupant age by 11 years. Thus, including just Delta-v and age as parameters in the stochastic simulation seems to be a reasonable first approximation.

In the stochastic simulation study, it was shown that the newly developed risk function, in particular the updated age effect, gives results that are more consistent with rib fracture risk estimated directly from NASS/CDS data. In general, the stochastic simulations predicted higher risk than the NASS/CDS did. The 50% rib fracture risk for the lateral stochastic simulations was estimated for a Delta-v 9 km/h (30-year-old) to 11 km/h (70-year-old) lower than the risk for NASS/CDS data. For the frontal stochastic simulations, the corresponding values were 29 km/h (30-year-old) to 17 km/h (70-year-old). Hence, all simulation results predicted higher risk than the NASS/CDS estimates. The stochastic simulations are defined using a few parameters, with distribution based on NASS/CDS. Most likely there are many additional parameters significantly influencing injury outcome, not reported in databases like NASS/CDS (simply because they cannot be measured) and thus cannot easily be included in stochastic simulations. In addition, as safety system parameters are proprietary information, these had to be estimated based on reverse engineering from US NCAP tests. That in combination with a sampling strategy not considering potential dependency of these parameters, makes it highly likely that the generic safety system will perform less optimal compared to systems in production vehicles.

It should also be noted that the rib fracture risk estimated from the NASS/CDS data should not be considered as an absolute truth. Several studies have shown that the true fracture rate is under-reported by as much as 50–70% when fractures are diagnosed using clinical CT (Crandall et al., 2000; Lederer et al., 2004; Schulze et al., 2013), and thus the NASS/CDS risk curves might underestimate the true fracture risk. This means that probably neither the injury risk from the stochastic simulations nor the real-life estimated risk is correct. However, comparing the stochastic simulation results evaluated using the newly developed risk function with the Forman smoothed risk function and the NASS/CDS estimated risk, the newly developed risk curve seems to estimate the age effect better than the Forman smoothened risk function.

The main effect on HBM rib fracture risk predictions of using the newly developed risk function instead of the Forman 2012 or the Forman smoothed risk functions will be a lower rib fracture risk predicted for younger occupants for the same level of rib strain. Out of the 36,560 people fatally injured in motor vehicle accidents in the United States during 2018, 6,087 were aged between 16 and 24 years (National Center for Statistics and Analysis, 2020). Kent et al. (2005) found that 75% of fatal injuries to younger drivers (16–33 years old) protected by both a seatbelt and an airbag in a frontal impact were to the head, whereas older occupants (65+ years old) crashed at lower Delta-v’s but were more likely to sustain a fatal chest injury. In order to reduce fatalities, for younger occupants a restraint system could apply a higher seatbelt restraint force in frontal impacts, in order to restrict head forward motion relative to the vehicle and thus avoid a hard head impact; for older occupants, a lower seatbelt restraint force in lower-severity accidents would be appropriate to mitigate chest injuries. That is, a plausible safety-system design solution would incorporate age-adaptive restraints. The newly developed risk function can be a useful tool in the design of such a restraint.

### Limitations

The rib fracture risk curve is based on failure strains obtained in 0.5 strain/s experiments and should therefore be used for strains obtained under similar strain rates. However, Katzenberger et al. (2020) found no statistically significant differences in failure strains between the 0.5 and 0.005/s strain rates tested. Experiments performed at yet higher loading rates may reveal if there is a rate effect to rib failure strain that need to be considered in future risk modeling.

Further, the experiments were tensile, and thus the developed risk function is only applicable to tensile strain, even though the strain experienced by ribs in motor vehicle accidents is not known. By using strain gages attached to the cutaneous side of PMHS’s ribs in an experiment simulating a belted frontal impact, Duma et al. (2005) demonstrated that the first principal strain was closely aligned to the axial strain (along the rib) and that a majority of ribs sustained tensile loading until fracture. Trosseille et al. (2008) measured the strain at PMHS’s ribs during different impact scenarios, ranging from frontal to lateral. The pattern of axial strain along the rib ranged from tensile to compressive and the distribution of strain was dependent on both loading direction and impacting object. Hence, if there are large shear or compressive rib strains predicted by an HBM, the resulting rib fracture risks obtained from the newly developed function (with tensile rib strains from the HBM simulation) might not reveal the full extent of the fracture risk.

There are many limitations with the stochastic simulations, of which some have been discussed above. In addition to using only one anthropometry, i.e., a model of an average male, only one initial posture was used, Further, injury risk age dependency was only modeled as change in ultimate strain, where in reality there are many other age related changes on both material and structural level.

## Conclusion

• A new strain-based, age-adjusted risk function that can be used to predict rib fracture risk together with finite element HBMs has been developed.
• The new fracture risk function indicates that human rib cortical bone failure strain is reduced by 12.2% per decade of aging.

• The new fracture risk function can be used directly within the existing probabilistic framework for estimating rib fracture risk.
• In stochastic frontal impacts the 50% risk of NFR2+ for a 30-year-old occupant was estimated at a DV of 60 km/h (Forman smoothed) and at 69 km/h with the newly developed risk function. For 70-year-olds the 50% NFR2+ Delta-v’s where 51 and 48 km/h using Forman smoothed and the newly developed risk function, respectively.
• In stochastic lateral impacts the 50% risk of NFR2+ for 30-year-olds was at Delta-v’s of 43 km/h (Forman smoothed) and 52 km/h (newly developed). For 70-year-olds the Delta-v’s were 35 and 36 km/h for Forman smoothed and the newly developed risk function, respectively.

## Data Availability Statement

The original contributions presented in the study are included in the article/supplementary material, further inquiries can be directed to the corresponding author/s.

## Author Contributions

K-JL: conceptualization, methodology, project administration, supervision, validation, and writing – original draft. AB: data curation, formal analysis, visualization, and writing – original draft. JI: conceptualization, formal analysis, visualization, and writing – original draft, reviewing, and editing. BP: conceptualization, supervision, resources, and writing – reviewing and editing. NL: conceptualization, supervision, resources, methodology, funding acquisition, and writing – reviewing and editing. All authors contributed to the article and approved the submitted version.

## Conflict of Interest

The authors declare that the research was conducted in the absence of any commercial or financial relationships that could be construed as a potential conflict of interest.

## Appendices

### Appendix A

Parametric expressions for fracture risk functions based on Weibull, log-normal, and log-logistic distributions are presented in Eqs A1, A2, and A3, respectively. Values for parameters α, β_0_, and β_1_ can be found in Table 1. LN() is the natural logarithm, exp() is the natural exponential function and erf() is the Gauss error function.

(A1) $$Weibullrisk\left(strain,AGE\right)=1-exp\left(-{\left(\frac{strain}{exp\left(\beta_{0}+\beta_{1}\cdot AGE\right)}\right)}^{\alpha }\right)$$

(A2) $$Log-normalrisk\left(strain,AGE\right)=\frac{1}{2}+\frac{1}{2}\mathrm{erf}\left[\frac{\mathrm{LN}\left(\mathrm{strain}\right)-\left(\beta_{0}+\beta_{1}\cdot \mathrm{AGE}\right)}{\sqrt{2}\cdot \alpha }\right]$$

(A3) $$Log-logisticrisk\left(strain,AGE\right)=1-\frac{1}{1+{\left(\frac{strain}{exp\left(\beta_{0}+\beta_{1}\cdot AGE\right)}\right)}^{\alpha }}$$

### Appendix B

The output statistics from the ANOVA analysis are presented in Table B1.

**TABLE B1:** ANOVA analysis results.

**Covariate**	**Coefficient**	**Df**	**Sum square**	**Mean square**	***F*-value**	***P* (>*F*)**
Age	−1.54E-04	1	4.44E-03	4.44E-03	6.00E+01	2.57E-10
Sex	8.88E-03	1	7.00E-05	7.00E-05	9.45E-01	3.35E-01
Age × sex	−1.18E-04	1	1.32E-04	1.32E-04	1.79E+00	1.87E-01
Residuals		54	4.00E-03	7.40E-05		

Tukey-Anscombe plots of residuals versus fitted values are shown in Figure B1.

Q–Q plots of survival model residuals versus the distribution assumptions are shown in Figure B2.

In Table B2 the QIs computed based on the relative sizes of the 95% confidence intervals are presented.

**TABLE B2:** Relative size of 95% confidence interval and thecorresponding quality index (QI) for Weibull, log-normal, and log-logistic distributions.

**Distribution**	**Age**	**Risk of injury (%)**	**Relative CI size**	**Quality index**
		5	0.423	Good
	25	25	0.281	Good
		50	0.237	Good
		5	0.431	Good
Weibull	50	25	0.255	Good
		50	0.177	Good
		5	0.464	Good
	75	25	0.289	Good
		50	0.215	Good
		5	0.334	Good
	25	25	0.260	Good
		50	0.253	Good
		5	0.279	Good
Log-logistic	50	25	0.175	Good
		50	0.158	Good
		5	0.293	Good
	75	25	0.218	Good
		50	0.196	Good
		5	0.283	Good
	25	25	0.238	Good
		50	0.241	Good
		5	0.248	Good
Log-normal	50	25	0.181	Good
		50	0.153	Good
		5	0.262	Good
	75	25	0.205	Good
		50	0.195	Good
